# Changes of activity and isoforms of alkaline sphingomyelinase (nucleotide pyrophosphatase phosphodiesterase 7) in bile from patients undergoing endoscopic retrograde cholangiopancreatography

**DOI:** 10.1186/1471-230X-14-138

**Published:** 2014-08-07

**Authors:** Rui-Dong Duan, Ulf Hindorf, Yajun Cheng, Per Bergenzaun, Mats Hall, Erik Hertervig, Åke Nilsson

**Affiliations:** 1 Gastroenterology & Nutrition Laboratory, BMC, B11, Department of Clinical Sciences in Lund, University of Lund, S-22184 Lund, Sweden; 2 Department of Gastroenterology, Skåne University Hospital, S-22185 Lund, Sweden

**Keywords:** NPP7, Alk-SMase, Bile, Patients, Cholangiocarcinoma, Liver diseases

## Abstract

**Background:**

Alkaline sphingomyelinase (NPP7) is an ecto-enzyme expressed in intestinal mucosa, which hydrolyses sphingomyelin (SM) to ceramide and inactivates platelet activating factor. It is also expressed in human liver and released in the bile. The enzyme may have anti-tumour and anti-inflammatory effects in colon and its levels are decreased in patients with colon cancer and ulcerative colitis. Active NPP7 is translated from a transcript of 1.4 kb, whereas an inactive form from a 1.2 kb mRNA was found in colon and liver cancer cell lines. While the roles of NPP7 in colon cancer have been intensively studied, less is known about the function and implications of NPP7 in the bile. The present study examines the changes of NPP7 in bile of patients with various hepatobiliary diseases.

**Methods:**

Bile samples were obtained at endoscopic retrograde cholangiopancreatography (ERCP) in 59 patients with gallstone, other benign disease, tumour, and primary sclerosing cholangitis (PSC). The NPP7 activity was determined. The appearance of the 1.4 and 1.2 kb products in the bile was examined by Western blot. The results were correlated to the diseases and also plasma bilirubin and alkaline phosphatase.

**Results:**

NPP7 activity in the tumour group was significantly lower than in the gallstone group (p < 0.05). The activity in the tumour plus PSC group was also lower than in gallstone plus other benign disease group (p < 0.05). Within the tumour group NPP7 activity was lowest in cholangiocarcinoma patients, being only 19% of that in gallstone patients. Bilirubin correlated inversely to NPP7 and was higher in the tumour than in the gallstone group. Western blot identified both the 1.4 kb and the 1.2 kb products in most bile samples. The density ratio for the 1.4/1.2 kb products correlated to NPP7 activity significantly. Two patients (one PSC and one cholangiocarcinoma) lacking NPP7 activity had only the 1.2 kb form in bile.

**Conclusion:**

NPP7 activity and the ratio of 1.4/1.2 kb products in bile are significantly decreased in malignancy, particularly in cholangiocarcinoma. The implications of the finding in diagnosis of cholangiocarcinoma and 1.2 kb product in hepatobiliary diseases require further investigation.

## Background

Alkaline sphingomyelinase is an intestinal brush border enzyme highly expressed in jejunum and ileum [1,2] and released into the lumen by bile salts and trypsin [3,4]. It is resistant to pancreatic and faecal proteases [3] and high activity can be detected in the faeces [5]. Purification and cloning showed that the enzyme is a novel member of nucleotide pyrophosphatase phosphodiesterase (NPP) family and is now called NPP7 [6,7]. NPP7 cleaves phosphocholine moiety from both exogenous and endogenous sphingomyelin (SM) in the gut and turns SM to ceramide. Ceramide is a lipid messenger that inhibits cell proliferation and induces apoptosis and may counteract cancer development [8]. Via its phospholipase C activity NPP7 also hydrolyses and inactivates platelet activating factor (PAF) [9], an important molecule with proinflammatory properties [10]. NPP7 has thus important roles in generation of sphingolipid signals, regulation of PAF functions and in the absorption and reutilization of choline in the gut [11]. Our recent study in NPP7 -/- mice has confirmed the key role of the enzyme in SM digestion and ceramide generation in the intestinal tract [12].

NPP7 activity was also identified in human bile but not in bile of other species including rat, mice, pig, rabbit and baboon [1,13,14]. Since in humans the activity was not detected in the mucosa of gallbladder, bile NPP7 was indicated to be derived from liver. This was confirmed later by identification of NPP7 mRNA in HepG2 cells [15]. Physiologically, bile NPP7 may contribute to a more efficient digestion of SM in humans, since in patients with ileostomy, most of fed SM is hydrolysed and absorbed [16], whereas in rodents, SM digestion is relatively slow, extended and incomplete [17]. We previously showed that dietary SM inhibited cholesterol absorption and this inhibition was also more obvious in rodents than in humans [16,18].

Several pieces of evidence suggest anti-inflammatory and anti-tumour roles of NPP7 [11]. NPP7 activities decrease in patients with colon cancer [19], longstanding colitis [20] and familial adenomatous polyposis [21]. Incubation of the enzyme with colon carcinoma HT 29 cells inhibits cell proliferation [22]. In dextran sulphate induced experimental colitis in rats, rectal installation of the enzyme alleviates colitis [23]. Hypotheses behind these effects have been linked to the generation of ceramide and to the inactivation of PAF [11,24]. The impact of NPP7 in liver cancer is a field that has not been extensively explored, although preliminary assay in human liver biopsies showed that the NPP7 level tended to be lowered in liver diseases predisposing to liver tumours [15].

Due to the alternative splicing, two isoforms of NPP7 have been identified in both colon cancer HT29 and liver cancer HepG2 cell lines [25,15]. The active full length NPP7 is a protein translated from 1.4 kb transcript consisting of 5 exons. The aberrant splicing form lacks exon 4 and the protein translated from it has no activity against SM [25]. Considering the intensive studies on NPP7 in the intestine, relatively little is known about the enzyme expressed in the liver. Liver is an important organ in terms of synthesis and secretion of SM [26] and probably PAF [27,28], two major substrates of NPP7. We therefore asked how the NPP7 activity in bile varies with different hepatobiliary diseases and whether the changed NPP7 activity or appearance of the 1.2 kb form in bile could be linked to these diseases, and thus be a potential disease marker. In the present study, we analysed NPP7 activity and the occurrence of two isoforms in bile from 59 patients undergoing diagnostic and/or therapeutic endoscopic retrograde cholangiopancreatography (ERCP).

## Methods

### Patients

The study was approved by the Regional Ethics Committee of Lund University (Nr 443/2008) and was conducted in agreement with the Declaration of Helsinki from 1975, as revised in 1983. Patients undergoing ERCP on clinical indications were asked about permission to take a bile sample for the study during the examination. Approved written consent after both oral and written information was obtained from all 59 patients. Patient characteristics are given in Table 1. Major patient groups were those with gallstones, tumours and primary sclerosing cholangitis (PSC). Primary outcome of the study was to test the hypothesis that patients with tumours or PSC have lower NPP7 activity and/or more frequent appearance of the 1.2 kb form than those with gallstones or other benign disease. Bile samples were collected during standard ERCP procedures. Briefly, the papilla was cannulated with a sphincterotome. A guide-wire was then introduced. When it was positioned in the bile duct, the sphincterotome was introduced further and bile was aspirated with a 10 ml syringe. The bile samples were immediately stored on ice and sent to the laboratory for sample preparation as described below.

**Table 1:** Age, sex and diseases of patients in groups

**Group**	**Gallstone**	**Tumour**	**PSC**	**Others**	**Total**
Number	29	13	10	7	59
Female	19	7	6	3	35
Male	10	6	4	4	24
Age range	33–85	60–79	20–82	53–83	20–85
Median age	67	66	48	67	66
Mean age	68.5	66.0	50.2	68.4	65.2

### Sample preparation

The bile samples were centrifuged at 14000 rpm for 10 min at 4°C to remove the undissolved materials. A small aliquot of the supernatant was saved as original bile for NPP7 activity assay and protein determination. The rest of the samples were subjected to protein precipitation by ammonium sulphate which was added step by step up to 40% saturation with stirring. The samples were kept stirring at 4°C for 1 h followed by centrifugation at 14000 rpm for 10 min. The precipitated protein pellet was dissolved in PBS buffer and stored at -20°C for Western blot analysis.

### NPP7 and bile alkaline phosphatase activity assays

The activity of NPP7 in the bile against SM was determined as described previously, using ^14^C- choline labelled SM as substrate [29]. Briefly, 5 μl of the original bile was incubated in 95 μl 50 mM Tris–HCl buffer containing 0.15 M NaCl, 80 pmole ^14^C-SM, 4 μg unlabelled SM, 10 mM taurocholate, 2 mM EDTA, pH 9.0 at 37°C for 30 min. After phase partitioning, an aliquot of the upper phase containing the cleaved ^14^C-phosphocholine was taken for liquid scintillation counting. The activity of NPP7 was expressed as the formation of phosphocholine in 1 h by per ml of bile and by per mg protein in the bile samples. Alkaline phosphatase (ALP) activity in the bile was determined using β-nitrophenylphosphate as substrate in 10 mM Tris buffer pH 10.0 as described previously [30]. Protein in the bile was assayed using a kit from Bio-Rad using albumin as a standard.

### Western blot for NPP7

Western blot was performed as described previously [23]. Normally 50 μg of the precipitated bile proteins were subjected to 10% SDS-PAGE and transferred to a nitrocellulose membrane. The membrane was probed with anti-NPP7 antibody (1:5000) obtained from R&D System (Abingdon, UK). After washing, the membranes were reacted with second antibody against rabbit IgG conjugated with horseradish peroxidase. The NPP7 bands were identified by ECL advance reagents and the remitted light was recorded on Fuji Image. A standard purified NPP7 from human bile was routinely loaded as a positive marker of the product of 1.4 kb transcript. The densities of the NPP7 bands and the ratio of the densities of the products from 1.4 kb/1.2 kb transcripts were analyzed by MyImage Analysis Software from Thermo Scientific.

### Clinical biochemical analysis of the serum

Bilirubin, alkaline phosphatase and γ-glutamyltranspeptidase (γ-GT) in plasma were analysed the day before or in the morning the same day as the ERCP were available in 58 patients and C-reactive protein taken at these times in 38 patients. The levels of bilirubin, ALP and γ-GT were determined at the Clinical Chemistry Laboratory, Skåne University Hospital, Lund Sweden on a Cobas 701 instrument using reagents from Roche (Roche Diagnostics, Mannheim, Germany). Reference values are 5–25 μmole/L for bilirubin, 0.60–1.8 μkat/L for ALP and 0.15–1.2 μkat/L for women and 1.2–1.9 μkat/L for men >40 years old.

### Statistical analysis

Statistical analysis was performed by soft program Graphpad Prism 5. Since the groups in comparison were independent of each other, and the values showed non-normal distribution, the significance of the differences between different groups were evaluated by Mann–Whitney test, whereas the correlation was analysed by linear regression test. P < 0.05 was considered to be statistically significant.

## Results

### Patients

Age and sex distribution in disease groups are shown in Table 1. The gallstone group comprises 29 patients. Common bile duct stones (CBDS) were removed at ERCP in 22 patients with gallstone disease, after a sphincterotomy had been performed. The remaining 7 patients all had a history of gallstone disease and 5 of them had previously undergone cholecystectomy. The clinical suspicion of CBDS was strong, but at ERCP no stones or only sludge was found. It was, however, considered likely that the stones had passed recently.

In the tumour group of 13 patients, 5 had cholangiocarcinoma, 1 had gallbladder cancer, 5 had pancreatic cancer, 1 had a hepatocellular carcinoma superimposed on an advanced PSC, and 1 had colon cancer with liver metastases.

The group designated “Others” consists of 7 patients, of which 1 was severely icteric and appeared to have severe alcohol related liver disease with cholestatic features, and 1 had severe icterus and a cholestatic profile for unknown reasons, combined with renal insufficiency. The other 5 were less severely ill. They underwent ERCP because of abdominal pain, moderately pathologic liver tests and in two cases widened common bile duct on ultrasonography. At ERCP 1 was considered to have a benign stricture of the common bile duct, and 1 had an irregular common bile duct for unknown reasons. Brush cytology was normal and no sign of malignancy appeared during a two year follow up.

### NPP7 activity

**Figure 1:**
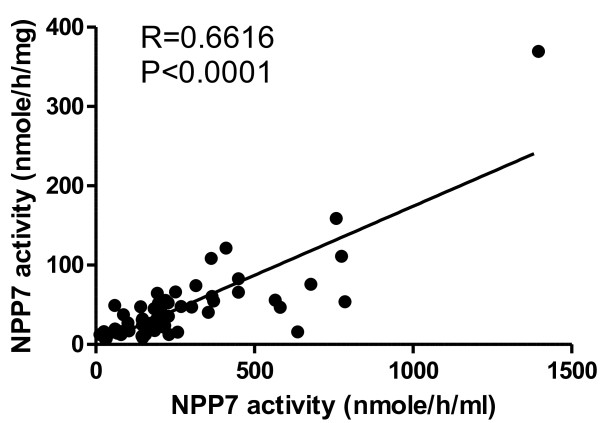
**Correlation of NPP7 activities in bile expressed as per ml and per mg sample protein.** NPP7 activity was determined using ^14^C-labeled sphingomyelin as substrate and the activities were expressed as the cleaved phosphocholine per ml of the bile and per mg of the bile proteins.

**Figure 2:**
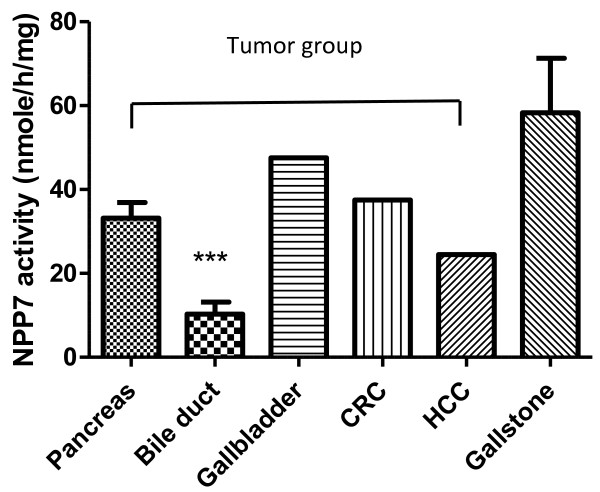
**NPP7 activities in patients in the tumour group.** The bile NPP7 activity was determined as described. N = 5 for pancreatic cancer and 5 for bile duct cancer (cholangiocarcinoma). N = 1 for gallbladder cancer, colorectal cancer (CRC) and hepatocellular carcinoma (HCC). The results are expressed as Mean of SE and ***P < 0.005 compared with Pancreas. For comparison, the values from gallstone patients are also presented (n = 29).

**Figure 3:**
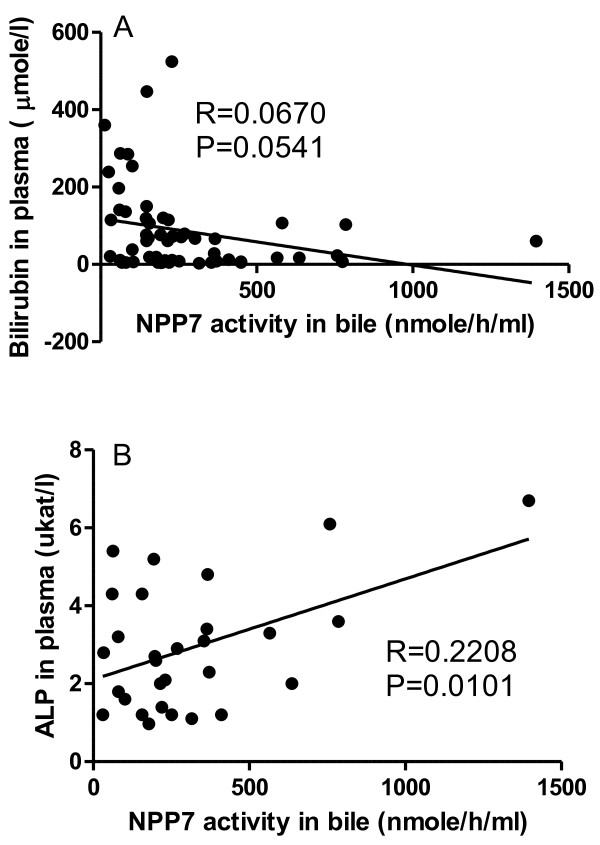
**Correlation of plasma bilirubin (A) and alkaline phosphatase (B) with NPP7 activity.** The plasma bilirubin and alkaline phosphatase (ALP) were assayed just before ERCP and bile NPP7 activity was assayed in the bile taken from ERCP. Correlation of the NPP7 activity to the plasma bilirubin in all samples is shown in **panel A**, and that of NPP7 activity to ALP in gallstone group is shown in **panel B**.

The results of the NPP7 analyses and cholestatic plasma parameters are shown in Table 2. Regardless if the NPP activity was expressed per ml bile or per mg protein in bile, the activity in the tumour group (T) was lower than in gallstone group (G) (p < 0.05). NPP7 in bile from the combined groups with gallstone and other (O) benign diseases (G + O) was also significantly higher than in the T and T plus primary sclerosing cholangitis (T + PSC) groups. Furthermore the NPP7 in the combined (T + PSC) was lower than in the gallstone group. As a general feature NPP7 enzyme activities per ml of bile and per mg bile protein correlated strongly (Figure 1). The NPP7 activity in tumour group is shown in Figure 2. Within this group, the NPP7 activity in the five patients with cholangiocarcinoma was significantly lower than in the five patients with pancreatic cancer (Figure 2). For comparison, the levels of NPP7 activity in gallstone group are also shown in the figure.When NPP7 values were correlated to plasma bilirubin, ALP, and γ-GT in all patients, a weak inverse correlation between bilirubin and NPP7 activity was identified (P = 0.054) (Figure 3A). Within the gallstone group there was a positive correlation between NPP7 activity and plasma ALP, although plasma ALP was only modestly raised in this group (Figure 3B). In the group of cholangiocarcinoma in Figure 2 bilirubin was 243 ± 172 μmole/L compared to 67 ± 40 μmole/L (p = 0.08) in the group of pancreatic cancers. C-reactive protein did not correlate to NPP7 activity (data not shown) and there was no indication that inclusion of some patients with pancreatitis or cholangitis related to stone in ductus choledochus significantly influenced group comparisons.

**Table 2:** NPP7 activity in the bile and the results of other clinical chemical analysis in patients in different groups

**Group**	**NPP7 nmol/h/ml**	**NPP7 nmol/h/mg**	**Bilirubin**	**ALP**	**GT**	**n**
Gallstone	311.6 ± 290.1	58.21 ± 69.93	54.59 ± 63.19	2.91 ± 1.60	6.69 ± 5.74	29
Tumor	164.9 ± 147.1*	25.16 ± 14.49*	161.2 ± 135.3**	12.2 ± 11.2**	14.15 ± 9.87*	13
PSC	184.4 ± 115.0	30.14 ± 26.97	56.67 ± 65.68	4.39 ± 3.00	5.06 ± 5.13	10
Others	312.6 ± 236.1	50.95 ± 31.09	124.9 ± 197.8	4.08 ± 3.52	4.75 ± 4.12	7
G + O	311.8 ± 277.3+	56.89 ± 63.92+	68.25 ± 103.4	3.14 ± 2.09	6.31 ± 5.47++	36
T + PSC	173.2 ± 131.4*	27.33 ± 20.45*	118.5 ± 121.9*	9.19 ± 9.69**	10.44 ± 9.31	23

The results are expressed as Mean ± SD. Statistical significance was determined by non-paired, two tails Mann–Whitney test. *P < 0.05, **P < 0.01 or less compared with gallstone group; + P < 0.05, ++ P < 0.01 or less, compared with tumor group. Significance was also found when gallstone + other benign diseases (G + O) was compared with tumour + PSC (T + PSC) for NPP7 and ALP, either per ml or per mg.

Two patients, a 73 year old woman with PSC and a 67 year old man with cholangiocarcinoma had extremely low levels of NPP7, 1.2 and 0.1 nmole/h/mg protein respectively. These patients had bilirubin levels of 22 and 156 μmole/L and ALP levels of 4.9 and 1.9 μkat/L respectively. ALP values in bile varied considerably between individuals but did not correlate to plasma bilirubin or γ-GT.

### Western blot for NPP7 isoforms

**Figure 4:**
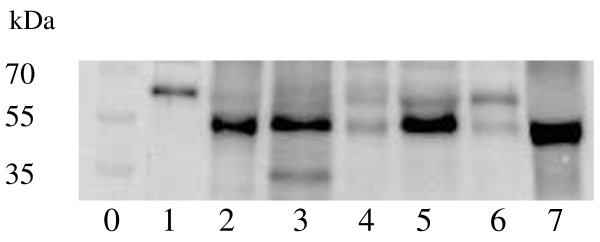
**A representative figure of Western blot showing the identification of the two isoforms.** The proteins in the bile were precipitated and 50 μg of proteins of each samples were subject to Western blot. Lane 0: standard proteins. Lane 1: purified human bile NPP7. Lane 2 to 7: samples from patients. As shown, two isoforms could be identified.

**Figure 5:**
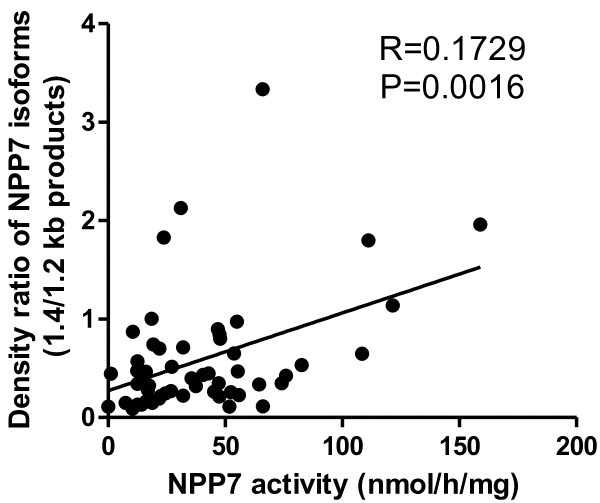
**Correlation of the ratio of protein bands from 1.4 and 1.2 kb transcript to NPP7 activities in all samples.** The densities of the two protein bands identified by Western blot were determined and the ratios of the two products in the same sample were analysed. The correlation of the ratio with NPP7 activities in all samples was determined.

To identify the occurrence of the inactive 1.2 kb product of NPP7, all the bile samples available (55 of 59 samples) were analysed by Western blotting. The antibody used can well identify these two isoforms as shown in Figure 4, which is a representative image. The main finding was that in most biles the 2 isoforms were observed with molecular masses corresponding to both the 1.4 kb and the 1.2 kb mRNA form. The density of the two bands were then determined and the density ratios of the isoforms for each bile sample were calculated. As shown in Figure 5 the ratio correlated significantly to the NPP7 activity in all samples. The two patients mentioned above with extremely low levels of NPP7 activity showed no band of the 1.4 kb product by Western blot.

## Discussion

The present study for the first time examines the activity and isoforms of NPP7 in the bile in humans with different diseases. The major findings are that although almost all bile samples contained considerable NPP7 activity, the activities were significantly decreased in the bile of patients with cholangiocarcinoma.

NPP7 is expressed in the intestinal tract and additionally in human liver [11]. NPP7 from human liver is released in bile, probably by bile salt action on the canalicular membrane, analogous to the release from intestinal segments by luminal bile salt perfusion [4]. Due to the generation of ceramide and inactivation of PAF, the enzyme has been considered to have anticancer properties [11]. Reduction of NPP7 level has been reported in colorectal cancer [19] and longstanding colitis [20], and an inactive isoform of NPP7 was identified in both colon cancer HT29 and liver cancer Hep G2 cell lines [15,25]. In the present study, the lowered NPP7 activity in bile of patients with cancers comparing with those with gallstones is in agreement with previous studies in human colon cancer samples [19]. The finding that most obvious reduction of NPP7 was found in cholangiocarcinoma patients is of particular interest. Cholangiocarcinoma is a cancer with increasing incidence, and the disease is considered incurable except for the few cases where surgical removal is possible. Due to the lack of an early marker, most cholangiocarcinomas are diagnosed at a stage when surgical cure is impossible, and the 5 year survival rate for the patients is almost zero [31,32]. The possibility that the great reduction of NPP7 in bile can be a marker in prediction of cholangiocarcinoma at early stage is attractive. It may be noteworthy that reduced NPP7 activity was also found in the patients with the chronic bile duct inflammation PSC, which has strong links to ulcerative colitis and risk for cholangiocarcinoma [33,34].

The occurrence of the aberrant NPP7 from the 1.2 kb mRNA in the majority of the bile samples is not a finding we expected, but interesting. It may indicate that appearance of the aberrant isoform cannot simply be used as a marker for malign hepatobiliary disease. The strong correlation between the ratio of 1.4/1.2 kb products and NPP7 activity supports our previous finding that the 1.4 kb product is active whereas the 1.2 kb form is not [25]. It also suggests that the secretion of the 1.2 kb product in bile was actually less affected than the secretion of the 1.4 kb product in the tumour and tumour plus PSC group. The result also argues against a nonspecific effect of cholestasis as explanation of the lower NPP7 activity in these groups. Interestingly two patients, a 73 year old woman with PSC and a 68 year old man with cholangiocarcinoma (Klatskin tumour) had almost immeasurable NPP7 activity but strong bands corresponding to the 1.2 kb and no band matching the 1.4 kb transcript. Although the aberrant 1.2 kb form has no activity against SM, this protein may have other functions that have not been identified. It is well known that alternative splicing is an important mechanism to produce isoforms with different, or opposite functions compared to the normal product [35,36].

NPP7 has 5N-glycosylation sites and defect glycosylation reduces the enzyme activity [37]. Since the identification of the 1.4 and 1.2 kb products in the present study was based on Western blot, one might ask whether the product with smaller molecular weight is derived from abnormally glycosylation. We consider this an unlikely explanation of the molecular mass difference. The 1.2 kb mRNA for the isoform has been shown to be expressed in liver cells and transfection of Cos7 cells with the 1.2 kb cDNA produces a protein band on Western blot similar as the one found in this study [15]. In addition, Dr. Yang LP in Nantong Cancer Research Institute, Jiangsu Province, China, also found the two bands on Western blot in human colon cancer tissues. In their experiment, pretreating colon cancer samples with glycosidase did not affect the size differences between the two bands on Western blot (personal communication). However, further studies to characterize sugar moiety of the two products in human bile is still necessary, as abnormal glycosylation has great impact on carcinogenesis [38].

In the present study, we also found a week positive correlation between bile NPP7 and plasma ALP within the gallstone group, suggesting that cholestatic stress may increase NPP7 secretion in bile. There was, however, no such correlation in whole patient population or within the tumour group which had higher ALP level than the gallstone patients. In the whole patient population there was rather a week inverse correlation between NPP7 and bilirubin levels, and NPP7 was lower but bilirubin higher in cholangiocarcinoma than in pancreas cancer patients. If cholestasis itself stimulates secretion of NPP7 into bile, other factors thus may act in tumour group to counteract this phenomenon. Although we earlier found a trend towards low NPP7 levels in liver biopsies from humans with liver disease [15], only one of the tumour patients in this study had known liver disease (hepatocellular cancer in a PSC liver). Pre-existing liver disease therefore does not explain the lower NPP7 values in the tumour group.

## Conclusions

In conclusion NPP activity was decreased with malign hepatobiliary diseases and lowest activity was associated with cholangiocarcinoma. Although the occurrence of both the normal 1.4 kb and the aberrant inactive 1.2 kb form of NPP7 in human bile is a general phenomenon, the NPP7 activity is derived from the 1.4 kb form. However, what determines the partitioning between the two forms, and the identity and function of the 1.2 kb form require further study.

### Limitations of the study

Although the results of the study are potentially important, a few limitations need to be noted. Since cholangiocarcinoma is not a common cancer disease, the number of the patients with cholangiocarcinoma in the present study is small. Further study in a large scale is required. In addition, as an ectoenzyme, bile NPP7 is expected to be expressed in the canalicular membrane of the hepatic cells, but the precise location of NPP7 in liver cells has not been clearly demonstrated. This is partly due to the fact that NPP7 has not been found in the liver of animals so far, and its levels are down regulated in cancer tissues. Immunohistochemistry study on normal and diseased human liver should be performed when possible. Finally we also lack the genetic analysis on the two patients in the study who lack NPP7.

## Abbreviations

ALP: Alkaline phosphatase; CBDS: Common bile duct stone; ERCP: Endoscopic retrograde cholangiopancreatography; γ-GT: Gamma glutamyl transpeptidase; NPP: Nucleotide pyrophosphatase phosphodiesterase; PSC: Primary sclerosing cholangitis; SM: Sphingomyelin.

## Competing interests

No competing interests with others.

## Authors’ contributions

RD: conception and design of the experiment, analysis of the samples, data interpretation, manuscript writing. UH: project organization, patient characterization, ERCP performance, data interpretation, manuscript revision. YC: Lab work organization, sample and data analysis. PB and MH: ERCP performance, patient characterization, manuscript revision. EH: project planning, manuscript revision. ÅN: project organization, patient characterization, data interpretation, manuscript writing. All authors read and approved the final manuscript.

## Pre-publication history

The pre-publication history for this paper can be accessed here:

http://www.biomedcentral.com/1471-230X/14/138/prepub
